# Breast and gynecologic cancer-related extremity lymphedema: a review of diagnostic modalities and management options

**DOI:** 10.1186/1477-7819-11-237

**Published:** 2013-09-22

**Authors:** Pankaj Tiwari, Michelle Coriddi, Ritu Salani, Stephen P Povoski

**Affiliations:** 1 Department of Plastic Surgery, The Ohio State University Wexner Medical Center, Columbus, OH 43210, USA; 2 Division of Gynecologic Oncology, Department of Obstetrics and Gynecology, Arthur G. James Cancer Hospital and Richard J. Solove Research Institute and Comprehensive Cancer Center, The Ohio State University Wexner Medical Center, Columbus, OH 43210, USA; 3 Division of Surgical Oncology, Department of Surgery, Arthur G. James Cancer Hospital and Richard J. Solove Research Institute and Comprehensive Cancer Center, The Ohio State University Wexner Medical Center, Columbus, OH 43210, USA

**Keywords:** Lymphedema, Vascularized lymph node transfer, Lymphography, Lymphadenectomy, Malignancy, Cancer surgery

## Abstract

Lymphedema remains a poorly understood entity that can occur after lymphadenectomy. Herein, we will review the pathogenesis of lymphedema, diagnostic modalities and the natural history of extremity involvement. We will review the incidence of upper extremity lymphedema in patients treated for breast malignancies and lower extremity lymphedema in those treated for gynecologic malignancy. Finally, we will review traditional treatment modalities for lymphedema, as well as introduce new surgical treatment modalities that are under active investigation.

## Background

### Definition, classification and pathogenesis of lymphedema

Along with the arterial and venous vasculature, the lymphatic system represents an important component of the circulatory system. Lymphatic channels primarily regulate the flow of fluid in the interstitium [1]. Under normal conditions, venous capillaries reabsorb 90% of the fluid in the tissues, and lymphatic channels absorb the remaining 10% of lymph fluid, proteins and other molecules [2]. Lymphatic fluid then passes to the regional lymph node basins. Ultimately, this fluid is transported back to the left subclavian vein to enter the venous system via the thoracic duct.

Lymphedema results from lymphatic insufficiency and inadequate lymph transport [3]. Decreased lymph transport causes an accumulation of protein-rich interstitial fluid, leading to distention, proliferation of fatty tissue and progressive fibrosis. Thickening of skin and hair loss may subsequently occur. Progressive lymphedema without adequate management can lead to functional impairment, compromised quality of life and physical deformity. Clinically, lymphedema is noted as swelling of the involved extremity. The head, neck, breast, or genitalia may also be affected [4-6].

Lymphedema is generally classified as either primary or secondary.

Primary lymphedema is related to congenital malformation of the lymphatic channels. It can result from any one of a number of disorders that may be sporadic or hereditary. The estimated prevalence of primary lymphedema is 1.15 in 100,000 persons under the age of 20 [5]. In children, the two main causes are Milroy disease and lymphedema distichiasis [3].

Secondary lymphedema is a consequence of surgical removal or damage to lymph nodes, post-radiation fibrosis of the lymph nodes, trauma, or infection [6]. Upper extremity lymphedema is commonly associated with the treatment of breast cancer. The degree of lymphedema correlates with the number of lymph nodes that have been removed and the extent of radiation treatment to the axillary region. Lower extremity lymphedema is seen in patients treated for gynecologic malignancy and prostate cancer, as well as melanoma and lymphoma [7]. Most patients develop lymphedema within three years of treatment [8]. In addition to cancer treatment-related lymphedema, side effects of advanced diseases, such as congestive heart failure, neurological and liver disease and end-stage renal disease can cause chronic lymphedema. An increase in the bariatric population has also seen an increase in lymphedema incidence [9]. Lymphedema caused by the parasite *Wucheria bancrofti* and transmitted by mosquitoes, remains the most common cause of lymphedema worldwide.

Unfortunately, no strategies employed to prevent the onset of lymphedema have proven fruitful to date. New clinical data suggest that some patients may have a primary predisposition to lymphedema; however, in these instances, lymphedema does not become clinically evident until after the occurrence of some secondary eliciting event [6]. Lymphedema tarda is defined as new onset lymphedema after the age of 35. It is often associated with an eliciting event such as trauma or infection. Once established, lymphedema may progress to a chronic condition. 'Chronic lymphedema’ is generally categorized as lymphedema persisting for more than three months. Prevalence estimates for chronic lymphedema are between 1.3 and 1.5 per 1,000.

At the cellular level, lymphedema is a complex interplay of lymphangiogenesis, inflammation, fibrosis and lipid metabolism [10]. Recent work has demonstrated that inflammatory responses may play a significant role in lymphedema pathogenesis. Lymphatic stasis results in CD4(+) T-cell inflammation and T-helper 2 (Th2) differentiation. Using mice deficient in T cells or CD4(+) cells, Avraham *et al*. have shown that the inflammatory response is necessary for the pathological changes of lymphedema, including fibrosis, adipose deposition and lymphatic dysfunction [11]. Such work carries the potential to identify better the molecular mechanisms underlying the progressive fibrosis associated with chronic lymphedema. Intervention at the signal transduction level may prevent and/or reverse the development of fibrosis that occurs in chronic lymphedema.

### Diagnosis of lymphedema

The diagnosis of lymphedema is typically made after a thorough history and physical examination. Initial stages of lymphedema begin with a soft pitting edema, usually in a unilateral extremity. Over time, chronic edema stimulates inflammatory changes in the subcutaneous space. These changes result in skin induration and thickening ultimately developing into a non-pitting edema with fibrotic changes of the skin and subcutaneous tissues.

The lymphedematous extremity is typically assessed in a clinic setting. Evaluation involves serial measurements over time to assess changes in the status of the involved extremity. If the contralateral extremity is uninvolved, measurements of this extremity are used as a control. There can be great inter-observer variability if the anatomic points of measurement are not standardized. At our institution, the upper extremity is measured as follows: middle finger, proximal to the proximal interphalangeal (PIP) joint and proximal to the metacarpophalangeal (MCP) joint; wrist proximal to the styloid; forearm four inches distal to the olecranon; elbow extended at crease; mid upper arm four inches proximal to the olecranon; axilla eight inches proximal to the olecranon. The lower extremity is measured as follows: instep proximal to the metatarsophalangeal (MTP); ankle proximal to the malleoli; calf six inches distal to the infra-patellar border; knee at the popliteal fold; mid-thigh six inches proximal to the superior patellar border; groin eight inches or ten inches superior to the superior patellar border. Associated pain and fatigue are also routinely recorded in the clinical assessment. Lastly, all patients are given the Lymphedema Breast Cancer Questionnaire (LBCQ) to fill out, representing a validated tool for the assessment of lymphedema symptoms [12].

When imaging is used, the most common modality for diagnosis is indirect radionuclide lymphoscintigraphy [13,14]. This procedure requires intradermal or subcutaneous injection of an appropriate radiolabeled tracer (^99m^Tc-antimony sulfide colloid or ^99m^Tc-labeled human serum albumin). Criteria for the diagnosis of lymphatic dysfunction include: (1) delayed, asymmetric or absent visualization of regional lymph nodes; (2) asymmetric visualization of lymphatic channels; (3) collateral lymphatic channels; (4) interrupted vascular structures; and (5) visualization of the lymph nodes of the deep lymphatic system. The presence of 'dermal back-flow’ is considered abnormal. It is interpreted to represent the extravasation of lymph fluid from the lymphatics into the interstitium as a result of lymphatic and/or venous hypertension [15].

Beyond lymphoscintigraphy, magnetic resonance imaging and computerized axial tomography have clinical utility. These imaging techniques permit objective documentation of the structural changes caused by lymphedema [16,17]. Recent advances in the magnetic resonance approach have improved the visualization of lymphatic vascular anomalies in both nonenhanced [18] and contrast-enhanced [19] applications.

At our institution, we have utilized a protocol similar to that described by Arrive *et al*. [20]. This protocol is similar to magnetic resonance (MR) lymphography of the retroperitoneal area and does not involve contrast injection. The MR lymphography utilizes a three-dimensional high spatial-resolution, fast spin-echo sequence. The main advantage of a three-dimensional isotropic MR lymphography protocol is to capture thinner section source images. Thinner images allow for the optimal processing of image data in order to obtain maximum intensity projection (MIP) images and multiplanar reformatted (MPR) images. MR lymphography also allows the potential advantage of a three-dimensional assessment of the extremity, thus potentially allowing for the calculation of extremity volumes.

The use of indocyanine green lymphography has emerged as an important imaging technique in the work-up of patients with upper extremity and lower extremity lymphedema. Indocyanine green lymphography allows pathophysiological lymphedema severity staging in real-time without radiation exposure. This technique has also been reported to be useful for intraoperative navigation for lymphatic surgery. With progression of secondary arm/leg/facial lymphedema, lymphography findings change from a normal 'linear’ pattern to an abnormal 'dermal backflow’ pattern. With this imaging, dermal backflow patterns can be visualized as a mild dermal backflow 'splash’ pattern, moderate dermal backflow 'stardust’ pattern, or severe dermal backflow 'diffuse’ pattern. Progression of the lymphography pattern may correspond to the pathology of the lymphatic system [21-25].

Bioelectric impedance spectroscopy analysis is an emerging diagnostic technique for the clinical evaluation of lymphatic edema. The technique uses resistance to electrical current in comparing fluid compartments within the body [26]. It has been considered as a cost-effective and reproducible method for evaluating patients with suspected lymphedema [26]. The technique allows for noninvasive quantification of extracellular fluid in the extremities. The technique is sensitive and reproducible and is likely to find increasing application in the early detection and management of lymphedema.

Finally, innovative biomarkers have also been developed to facilitate early-stage diagnosis. Microarray-based transcriptomics of human skin have been developed to identify patients with lymphedema. Such multi-variable biomarker panels should sensitively discriminate human lymphedema subjects from normal individuals [10].

## Review

### Breast cancer-related lymphedema

Breast cancer, including all cases of invasive and *in situ* carcinoma, is newly diagnosed in more than 296,000 women each year in the United States [27]. These women subsequently undergo treatment that can include surgical intervention, chemotherapy, radiation therapy, anti-estrogen therapy and/or targeted therapy [27]. Treatments are effective, with almost three million survivors of invasive breast cancer currently living the United States [28]. Nevertheless, breast cancer survivors may experience postoperative and long-term complications as a result of their treatment.

**Figure 1:**
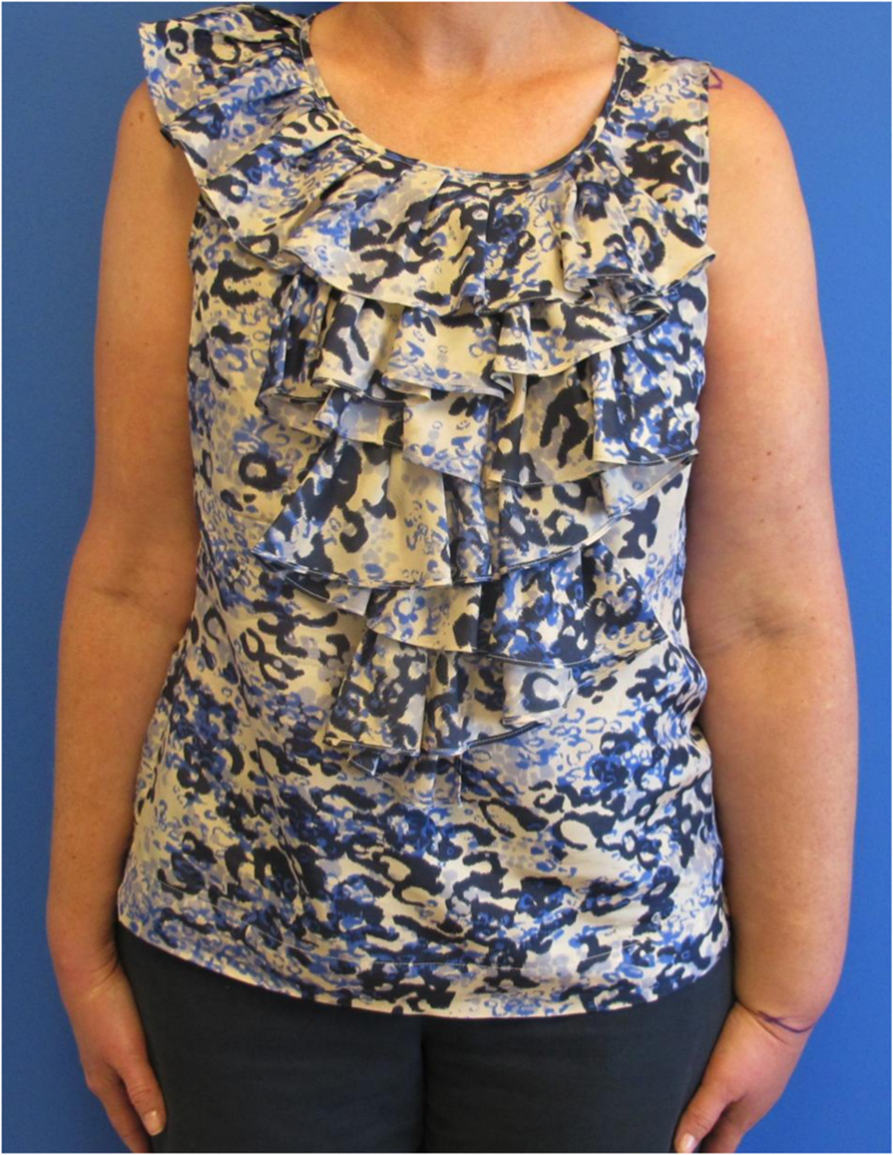
Left upper extremity lymphedema in a patient treated for breast cancer with modified radical mastectomy and axillary radiation treatment.

Lymphedema of the upper extremity is a well-recognized, long-term complication of either axillary lymph node dissection (ALND), or even of sentinel lymph node (SLN) biopsy alone [28]. The reported incidence of breast cancer related lymphedema with ALND varies widely, ranging from 2% to 56% [29-34]. Reports have demonstrated decreased quality of life outcome measures as well as psychosocial difficulties that may include body image disturbances, permanent uncertainty and adverse effects on relationships [35-37]. Further, lymphedema can interfere with activities of daily living by causing a restriction in range of motion, pain, increased skin tension, recurrent infection, extremity swelling and the patient’s perception of the feeling of heaviness in the affected extremity [38-40]. Impairment of function affects the ability to work and participate in sporting activities [38]. Skin changes, such as thickening and hair loss, may also occur. While patients may develop lymphedema at any time following their breast cancer treatment, 70% of affected individuals report that they have onset of symptoms within one to two years [31] (Figure 1).

Multiple staging systems exist regarding lymphedema of the upper extremity [41,42]. The International Society of Lymphology describes three stages of lymphedema. The staging is as follows: stage 0, latent condition with no evident swelling but impaired lymph transport; stage 1, early accumulation of fluid that subsides with limb elevation; stage 2, limb elevation alone does not reduce swelling and pitting may or may not be present; and stage 3, lymphostatic elephantiasis. These stages only refer to the physical condition of the extremity; a more detailed staging system needs to be developed to include pathology [42].

Risk factors for lymphedema development have been extensively studied and are widely reported in the literature. In relation to the breast cancer itself, a larger tumor size [43] and location in the upper outer quadrant [44] have been reported as risk factors. The Iowa Women’s Health Study of 1,287 patients found that a more advanced tumor stage, larger number of excised nodes and having tumor positive nodes were positively associated with lymphedema development [31]. Physical functioning was also found to be significantly lower in patients with cancer metastasis [45].

The wide range of reported incidence of breast cancer-related lymphedema is largely due to variations in treatment regimens, with some therapies having significantly higher rates of lymphedema development [31,32,43,46-48]. In a study of 516 patients, Veronesi *et al*. showed a decreased incidence of lymphedema in patients receiving SLN biopsy compared to those receiving ALND [33]. More recently, the National Surgical Adjuvant Breast and Bowel Project (NSABP) B-32 trial examined breast cancer related lymphedema at a three-year follow up of 1,975 patients with ALND and 2,008 patients with SLN biopsy. They concluded that lymphedema incidence increased with ALND when compared to SLN biopsy (14% versus 8%) [34]. Further, as the number of lymph nodes removed increases, so does the risk and severity of lymphedema [30,49]. Radiation to the breast or to the axillary lymph nodes also increases the risk and severity for lymphedema [30,32,49]. The combination of ALND and radiation has a higher incidence for lymphedema development than with either treatment alone [30]. Gartner *et al*. studied the effect of chemotherapy by examining different combinations of surgery and radiation therapy, with and without chemotherapy [38]. These authors have demonstrated that regardless of treatment regimen, chemotherapy increases the risk of lymphedema development [50]. It should be noted that an important factor associated with the wide range of reported lymphedema incidence is the subjective nature of the diagnostic criterion that is used for lymphedema. Some studies rely on >1 cm of extremity circumference difference between extremities while other studies rely on 'subjective symptoms’ as the chief diagnostic criterion.

Breast cancer reconstruction has been implicated as a possible contributor to development of lymphedema. However, studies have demonstrated that these concerns are unfounded [51,52]. Interestingly, a recent study has shown that breast reconstruction is associated with decreased rates of lymphedema development [53]. Card *et al*. showed that patients who did not undergo reconstruction, either with autologous tissue or implant based, were significantly more likely to develop breast cancer related lymphedema, 9.9% versus 3.7% [53].

Patient factors associated with breast cancer related lymphedema include body mass index (BMI) and age [30,31,45,54-56]. The Iowa Women’s Health Study found that a higher baseline BMI, larger waist and hip circumference and a poorer general state of health were positively associated with lymphedema development [31]. Ridner *et al*. report that patients with BMI >30 at the time of treatment are 3.6 times more likely to develop lymphedema [54]. The effect of age is more controversial. In a survey study of 3,253 patients, Gartner *et al*. reported that younger age was significantly related to more severe symptoms [38]. Conversely, Park *et al*. found that upper extremity function correlated to age, with older patients having poorer scores on the Disabilities of Arm Shoulder and Hand outcome measure [45]. These findings were corroborated by Pezner *et al*. who found the incidence of lymphedema was 25% at age greater than 60 years and 7% at age less than 60 years [57]. Additional lifestyle risk factors that have been reported include a sedentary lifestyle [58].

Some have proposed that patient factors and cancer treatment can only partially explain the risk of lymphedema development and that inherited genetic susceptibility must therefore play a role [59]. In one study of 120 patients, those with SNPs in the receptor genes, VEGFR2, VEGFR3 and RORC, were significantly more likely to have developed lymphedema [59]. Genetic predisposition must be further studied to clarify this possible association.

While risk factors have been identified, the mechanism by which they contribute to lymphedema development is less clear. Surgery physically alters lymphatic channels, decreasing the ability to drain lymphatic fluid and causing accumulation of fluid and tissue protein [31]. Radiation increases endothelial proliferation and fibrosis. Tumors induce an inflammatory state, and chemotherapy and radiation can further exacerbate this host-driven inflammatory response [60]. Advanced age has been postulated to increase the risk of lymphedema due to a decrease in the number of lymphovenous anastomoses that may open in response to increased extremity interstitial pressure, thereby reducing compensatory mechanisms. While many hypotheses exist, further research is needed to clarify the pathophysiology of breast cancer related lymphedema development.

Although no risk reduction strategies have been definitively substantiated, strategies include the avoidance of needle sticks and blood pressures in the ipsilateral upper extremity and prophylactic actions, such as wearing compression garments during air travel [47]. Prospective surveillance models have been proposed to identify lymphedema and implement treatment at an earlier stage [61]. Advocates of this program believed that earlier detection and treatment would decrease the need for intensive rehabilitation and also be cost-effective. Using the prospective surveillance model with interval screening, the cost to manage early-stage lymphedema per patient per year was found to be $636.19, compared to the cost to manage late-stage lymphedema in a traditional referral based model which was $3,124.92 [61].

Among breast cancer patients, a general lack of knowledge regarding lymphedema risk and risk reduction practices exists [62,63]. Kwan *et al*. conducted a survey study of 389 patients with invasive breast cancer to determine their level of lymphedema awareness. They found that on a scale of 0 to 7, the median lymphedema awareness score was 4. Further, older age was significantly associated with a lower score [62]. Other studies have shown that increased knowledge of lymphedema correlates with higher risk for development [64,65]. However, those patients with lymphedema who received adequate information had significantly reduced symptoms [64]. Nurse assisted education and exercise have been shown to decrease the degree of lymphedema [47,66]. Programs have been implemented nationwide to increase patient knowledge [67]. The American Lymphedema Framework Project is a national initiative developed under the leadership of clinical experts and researchers in the field of lymphedema. While the project has multiple goals, they have prioritized developing and providing appropriate practice-based lymphedema educational programs for breast cancer patients [68].

### Gynecologic oncology perspective on lower extremity lymphedema

The American Cancer Society estimates that there are more than one million gynecologic cancer survivors currently living in the United States [69]. As this number continues to grow, more awareness of long-term complications related to the disease and its treatment are gaining attention. Lower extremity lymphedema (LEL) has been largely understudied and is one of these unintended consequences. Currently, data on LEL as a result of gynecologic malignancies has been limited to retrospective studies and has been hindered due to a lack of standard diagnostic evaluations and assessments, making the diagnosis elusive [70,71]. Furthermore, co-morbid conditions, such as deep venous thrombosis, congestive heart failure, and medications, may cause lower extremity swelling making evaluation for LEL more challenging [72].

**Figure 2:**
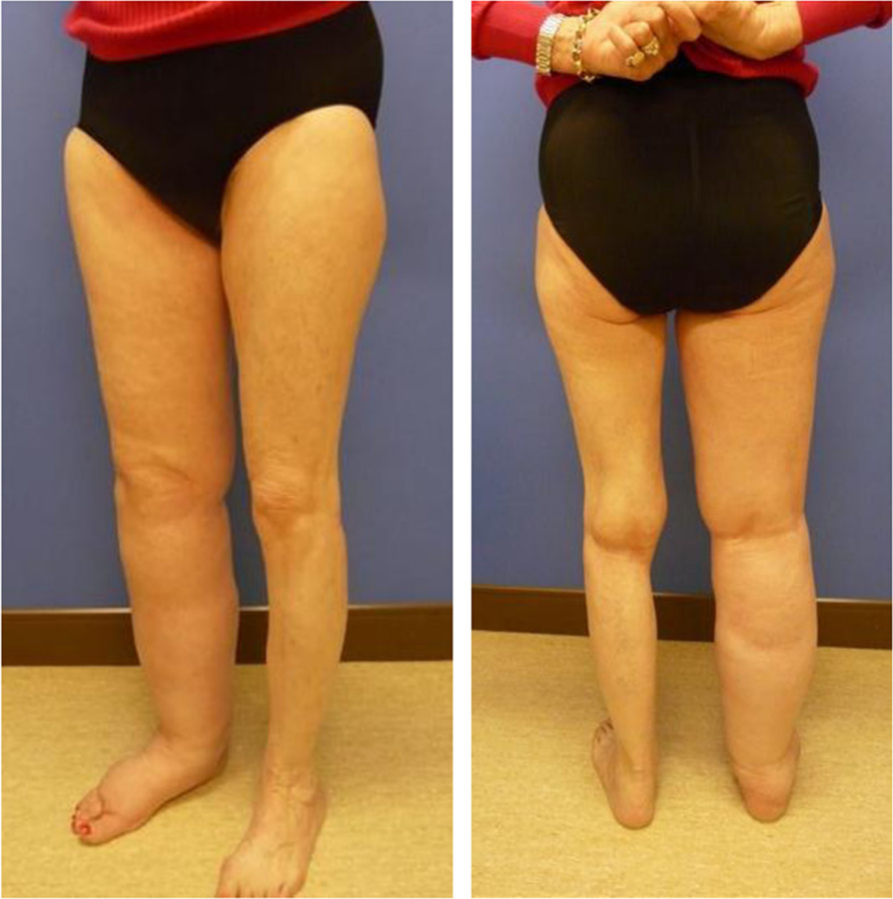
Right lower extremity lymphedema in a patient treated for endometrial cancer with lymphadenectomy and radiation treatment.

However, when LEL is diagnosed, the impact on patients can be substantial, both physically and psychologically. After treatment for gynecologic malignancies, LEL has been reported to affect a woman’s ability to function, including a decreased ability to perform activities of daily living and loss of work [73-75]. Additional physical consequences of LEL include leg discomfort secondary to heaviness, pain and skin tightness, as well as sexual difficulties [74,76,77] (Figure 2). In regard to psychological effects, gynecologic cancer survivors with LEL consistently report a decreased quality of life and self-confidence as well as increased anxiety and depression [73,74,78].

As cancer and its treatment are the most common cause for the development of secondary lymphedema in the United States, understanding its true impact is necessary [79]. In patients who have been treated for gynecologic malignancies, LEL risks may be categorized in the following groups: (1) preoperative factors (for example, genetic predisposition, obesity, age, race, nutritional status); (2) intraoperative factors (for example, number of lymph nodes removed, location of nodal dissection and procedures performed); and (3) postoperative factors (for example, adjuvant treatment, limb infection/trauma). Identification of risk factors and risk stratification may help gain insight into preventive measures and early detection/intervention for patients who are at increased risk for LEL [79]. Therefore, the need to gain insight into this condition is imperative. The purpose of this section is to provide a review of the current literature and overview of LEL in women with gynecologic cancers.

### Groin dissection and vulvar cancer

The primary management of vulvar cancer consists of radical surgical resection of the vulvar mass and groin or inguino-femoral lymph node dissection. As groin dissection is the lower extremity counterpart to axillary lymph node dissection, this procedure is correlated with the highest risk of LEL in gynecology, ranging from 25% to 67% [75,80-82]. Therefore, although it only affects an estimated 3,500 women per year in the United States, this treatment-related morbidity has been most extensively studied in vulvar cancer compared to other gynecologic cancers [83].

#### Risk factors and risk reduction

Consistent with other disease sites, one report noted that the removal of a higher number of lymph nodes, particularly more than six, was associated with a higher likelihood of LEL development [84]. Other factors increasing the risk of LEL include development of infection, and, interestingly, staple closure of the incision [80,81]. Other suggested risk factors include older age and diabetes [81]. Furthermore, high output from the groin drain was associated with higher risk of LEL and wound breakdown [81].

Efforts to reduce the rates of LEL in patients undergoing surgery for vulvar cancer have also been conducted. One of the largest impacts in reducing morbidity was seen with the incorporation of a three-incision technique when compared to the en bloc resection. This surgical modification, along with sparing of the saphenous vein, has significantly reduced the rate of LEL as well as other morbidity from this procedure [81,82,85]. In further efforts to reduce the association of surgical complications, particularly LEL, a randomized prospective trial using suture closure with or without the addition of fibrin sealant following groin dissection was evaluated in women undergoing surgery for vulvar cancer [80]. Unfortunately, rates of LEL based on limb measurements were high, exceeding 60% in both groups, and the use of fibrin sealant did not reduce LEL, leading to the need for alternative measures [80].

An area gaining rapid interest is the use of SLN biopsy in place of a complete lymph node dissection. Based on the data in the breast cancer literature, SLN biopsy has been successfully used in select cases with a reduction in surgical morbidity without compromising oncologic outcomes [86]. SLN biopsy has been shown to be a safe alternative to a full groin dissection in women with vulvar cancer, with similar groin recurrence rates [87,88]. More impressively, the rate of LEL in the SLN biopsy group was 1.9% compared to 25% in those undergoing a full dissection and improved surgical related outcomes [88,89]. As these methods become more commonly utilized and evaluated, the true impact of SLN biopsy may result in a change in the standard of practice, significantly reducing rates of LEL from the treatment of gynecologic malignancies.

#### Pelvic lymph node dissection and cervical, uterine and ovarian cancers

For malignancies involving the cervix, uterus and ovaries, primary therapy consists of surgical intervention with removal of the primary organ involved and the adjacent structures, along with a pelvic lymph node dissection (PLND) +/- para-aortic lymph node dissection [71,72,90]. Though less studied than in women undergoing groin dissection for vulvar cancer, LEL as a consequence of PLND has also been described. Retrospective studies report that the incidence of LEL in this group of patients ranges from 2.4% to 41% and has been shown to be significantly higher than in the general population [71,72,90-96].

#### Risk factors and risk reduction

Similar to groin dissection, the extent of lymph node dissection has consistently been shown to correlate with the development of LEL, although the number of lymph nodes ranges from 10 to 31 lymph nodes [71,72,92,97]. Another constant factor is the removal of the circumflex iliac nodes during a PLND, which breeches the groin lymph nodes and would expectantly result in higher rates of LEL as seen with the treatment for vulvar cancer [92,93,98,99]. Although not universal, a majority of studies report increased risk of LEL following surgery when compounded with radiation therapy [71,91,99-101].

Exacerbating factors for LEL are similar to those reported in upper extremity edema and those associated with groin dissection and include prolonged activity/standing and heat exposure [71]. However, with regard to other potential risk factors, such as age, retrospective studies have shown variable results [72,92,102]. Although obesity is independently associated with LEL, reports in gynecologic cancers have been less reliable [71,72,92,94,103].

Efforts to reduce the development of LEL secondary to PLND have been less extensively evaluated than its groin counterpart. In this forum, Fujiwara and colleagues evaluated the use of omentoplasty/omentopexy into the retroperitoneal nodal basins. As a result of this surgical procedure, the authors noted reduced rates of lymphedema and lymphocele in women with cervical and endometrial cancers compared to historical controls [104]. Another surgical modification, although most research has been conducted in vulvar cancer, is the role of SLN biopsy in place of PLND. Although larger studies are ongoing, the use of SLN biopsy mapping for cervical and endometrial cancer has demonstrated promising preliminary results [105,106]. As these methods are evaluated further and are more commonly utilized, the true impact of SLN biopsy may result in a change in the standard of practice, significantly reducing rates of LEL from the treatment of gynecologic malignancies.

### Diagnosis of lower extremity lymphedema

There are a multitude of factors limiting the understanding of LEL from gynecologic malignancies, regardless of the inciting cause of LEL (groin dissection or PLND). First, there is a lack of awareness of LEL, by both providers and patients, resulting in disregard of the condition or a delay in referral for treatment [70,71]. Secondly, inconsistent measurement techniques and lack of definition criteria have further hindered the ability to diagnose LEL [71,107]. Use of limb volume measurement with water displacement has been used; however due to feasibility issues, it is often limited to certain environments. Many studies have utilized lower limb circumferential measurements at pre-designated intervals comparable to assessments used in the literature regarding upper extremity lymphedema evaluations [108,109]. One of the most commonly reported tools is the use of subjective diagnosis based on the presence of symptoms. Carter and colleagues recently demonstrated that the Gynecologic Cancer Lymphedema Questionnaire (GCLQ) may be used as an effective tool to identify survivors with LEL after gynecologic cancer treatment [79].

In addition to direct measurements and questionnaires, imaging has also been evaluated. Although not practical for routine use, Lu *et al*. recently reported the presence of increased lymphatic vessels in affected extremities using MR lymphography to evaluate LEL secondary to gynecologic malignancies [110,111]. Regardless of which method is used, the need for improved and consistent diagnostic methods is apparent. Another complicating factor regarding diagnosis of LEL is the variable time of onset. Most studies report a diagnosis of LEL within the first year (median four to six months) after treatment, with 5% diagnosed within the first month [71,72,91,111]. However, long-term assessment should be performed as up to 20% of LEL cases are diagnosed after the first year [70,100].

### Future directions for lower extremity lymphedema

As awareness is increasing, efforts to address all aspects of LEL secondary to gynecologic cancers are improving. The incorporation of quality of life assessments, particularly those that focus on LEL, in gynecologic oncology studies will help develop our understanding of the impact of this condition. Understanding the incidence and developing a definition and consistent measure for LEL is critical. Currently, the Gynecologic Oncology Group (protocol 244) is conducting a prospective trial using a standardized objective limb measurement as well as subjective measurements/quality of life assessments in women undergoing surgery for vulvar, cervical or uterine cancers. Although the results will not be available for some time, this study is providing overdue attention to an important issue in survivors of gynecologic malignancies.

### Treatment modalities for lymphedema

When lymphedema is diagnosed, regardless of disease site, management is extremely variable, and currently there are no standard recommendations [70]. This lack of information results in inadequate or delayed management and, most likely, contributes to the decreased quality of life experienced by patients with LEL [70,71,74].

The state-of-the-art therapeutic approach to lymphedema relies upon physiotherapeutic techniques. Complex decongestive physiotherapy (CDPT) is designed to reduce limb volume and maintain the skin health and may stimulate lymphatic transport and facilitate the removal of retained interstitial proteins [112]. CDPT relies on a lymphatic-specific massage technique termed manual lymphatic drainage (MLD) [113]. A mild degree of manual tissue compression enhances filling of the cutaneous lymphatics and improves dilation and contraction of the lymphatic vessels. MLD may also recruit pathways for lymph flow and enhance the development of accessory lymph pathways.

In addition to MLD, the CDPT approach includes skin care, exercise and external compression. Short stretch bandaging compression creates a multilayer compartment that augments lymphatic contraction and flow [114]. During active tissue compression, abnormally increased ultrafiltration is reduced leading to improved fluid reabsorption. Once edema volume has decreased, maintenance of the therapeutic benefits will require fitted elastic garments. Nocturnal compression may also be required. Relatively inelastic sleeves and garments that transmit 40 to 80 mm Hg of compressive pressure will prevent fluid re-accumulation after successful CDPT. Garments must be fitted properly and replaced every three to six months.

While CDPT benefits the majority of patients with lymphedema, the interventions are labor-intensive, time-consuming and expensive. The potentially uncomfortable and visible garments may adversely affect the patient’s quality of life. In addition, the interventions are not uniformly successful. Most patients achieve adequate edema control, but some may require additional interventions [70]. Intermittent pneumatic compression has also been shown to augment the decompressive effects of standard therapies.

### Principles of the surgical management of lymphedema

Multiple surgical interventions for the treatment of lymphedema have been described, and any surgical intervention must be premised on well-defined indications. The typical surgical candidate has failed conservative therapy with an increase in the size and weight of the extremity and an impairment of extremity function. Patients who are recalcitrant to compression techniques may experience recurrent lymphangitis. In our practice, patients who have experienced three or more episodes of lymphangitis per year requiring the use of either oral or intravenous antibiotics are candidates for surgical intervention. Fibrofatty replacement of the subcutaneous tissue may worsen lymphatic fluid return and may limit the potential for successful surgical intervention. Although the gold standard for management of extremity lymphedema remains medical and includes decompressive measures, surgical treatments, including excision, lymphaticovenular bypass and vascularized lymph node transfer, are under active investigation.

Excisional techniques have been used since the 1910s and include debulking and liposuction [114]. Debulking surgery involves removal of lymphedematous adipose tissue down to fascia, followed by skin grafting or primary closure after elevation of skin flaps [114,115]. While earlier reports of this technique demonstrate its suboptimal outcomes and high complication rates, more recent reports show debulking can have good functional outcomes and minimal complications [115-120]. Salgado reports a 21% volume reduction using surgical excision in the upper extremity at more than one year post-operation [115]. Liposuction has been utilized more recently to remove subcutaneous lymphedematous fat and has proven to be effective for volume reduction [121-128]. In their study of 37 patients, Damstra *et al*. reported a 118% volume reduction of the upper extremity at one year following suction-assisted lipectomy [121]. Long-term results have also been reported with an upper extremity volume reduction of 101% at five years [122]. An advantage, aside from volume reduction, is increased skin blood flow which may decrease the incidence of cellulitis [124]. The most common disadvantage of this method includes transient numbness. Debulking surgeries, including excision and liposuction, effectively reduce lymphedematous volume, but may have the potential to violate the remaining, functional lymphatic structure. As a result, compression therapy is often necessary on a long-term basis.

Lymphaticovenular bypass is another surgical treatment of lymphedema. Supermicrosurgery with the aid of high power microscopy has been used to anastomose lymphatic channels to subdermal venules of less than 0.8 mm in diameter [114,129-134]. In 'microsurgical lymphaticovenous anastomosis’, lymphatic vessels with surrounding tissue are inserted into a vein (>2 mm), and anastomosis site thrombosis is inevitable when venous reflux occurs. In 'supermicrosurgical lymphaticovenular anastomosis’, a lymphatic vessel is anastomosed to a venule or smaller vein (approximately 0.5 mm) in an intima-to-intima coaptation manner and can prevent anastomosis site thrombosis even when venous reflux occurs [129-140]. This method is based on two concepts. Firstly, subdermal lymphatics are less affected by lymphedema and can be used for bypass. Secondly, pressure in subdermal venules is low and, therefore, venous backflow is minimized [130]. Chang prospectively utilized this technique in 20 patients with upper extremity lymphedema [130]. He created two to five anastomoses for each patient. Nineteen patients reported symptom improvement following surgery, and a mean volume reduction of 35% was seen at one-year post-operation [130].

An alternative method has been proposed by Campisi *et al*. The technique consists of anastomosing lymphatic vessels to a collateral branch of a main vein and confirming the functionality of the valvular apparatus. A functional valve will allow for unidirectional lymphatic flow, thereby limiting the potential for reflux of blood and anastomotic thrombosis. Healthy-appearing lymphatics found at the site of surgical operation are directly introduced into the vein by a U-shaped stitch and then fixed to the vein cut-end by additional stitches between the vein border and the perilymphatic adipose tissue [41]. In the largest retrospective study of 1,800 patients over 10 years, Campisi demonstrates a volume reduction of 67% and notes that 85% of patients were able to discontinue conservative treatment modalities [134]. Disadvantages to this procedure are that it is technically challenging and results are currently unpredictable [140]. Advantages of lymphaticovenular bypass include minimal invasiveness and a low rate of complications [131].

**Figure 3:**
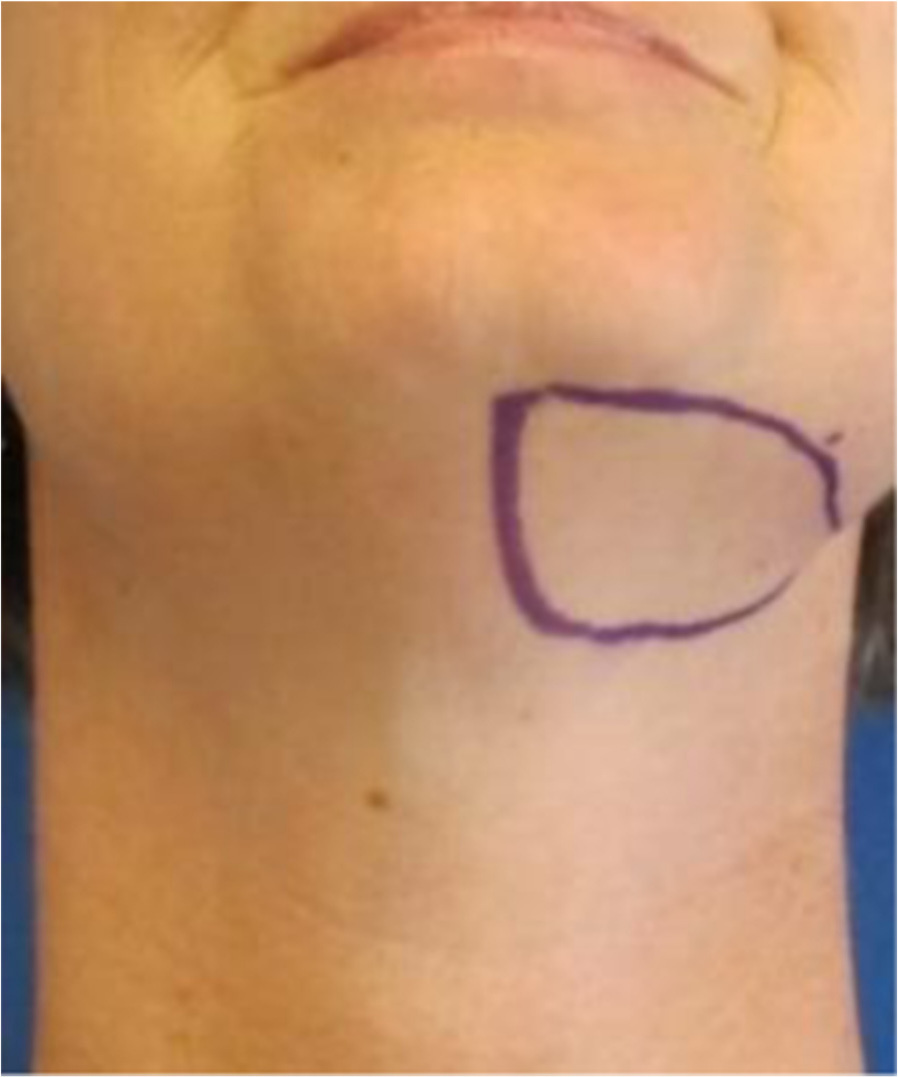
**Design for submental lymph node flap based on the submental vessels branching from the facial vessels.** The flap design is identical to a submental flap as used for head/neck reconstruction.

**Figure 4:**
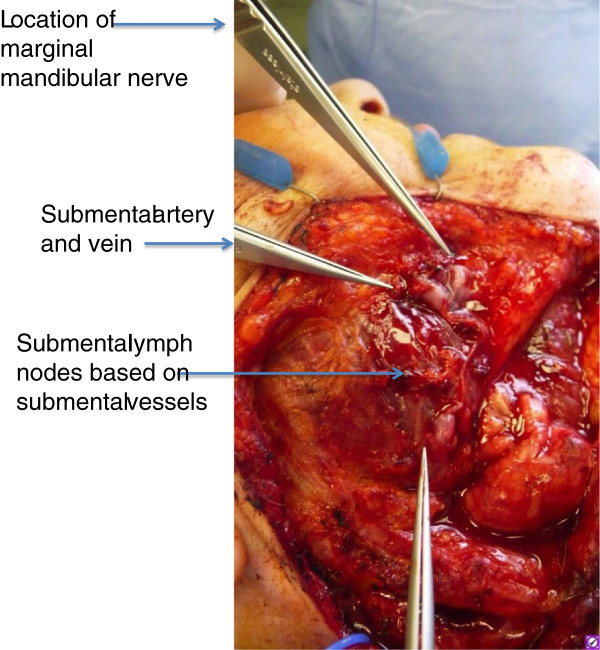
**Intraoperative view of cervical lymph node flap** (**level I**) **identifying submental vessels and facial nerve.**

**Figure 5:**
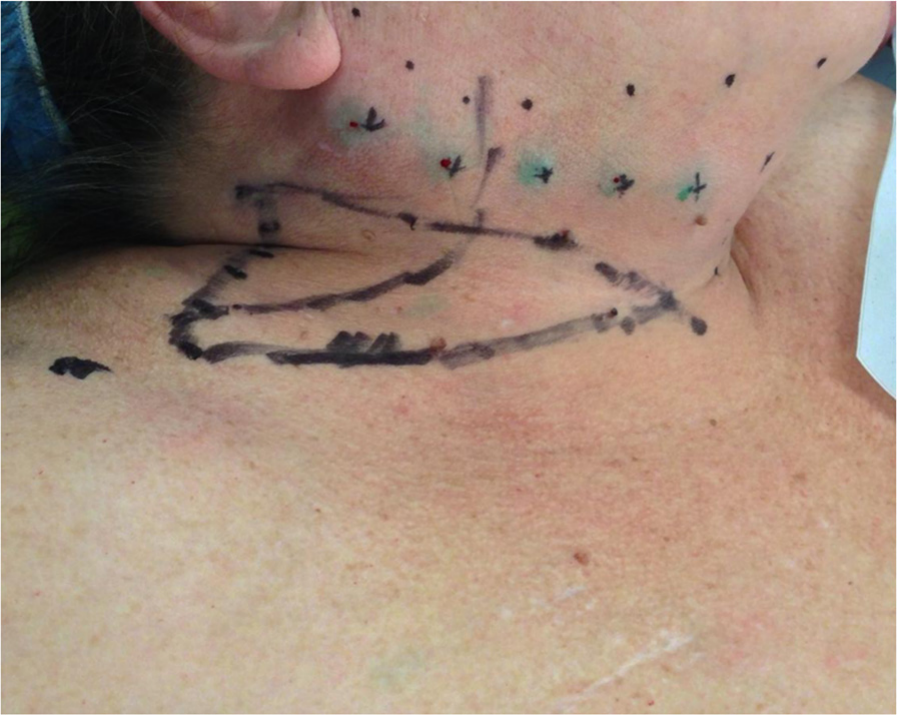
**Design of a planned cervical lymph node flap (level V) based on transverse cervical vessels.** Depicts sites of indocyanine green injection for intraoperative lymph node identification. The flap is based on the transverse cervical vessels located at the junction of the medial and middle thirds of the clavicle.

**Figure 6:**
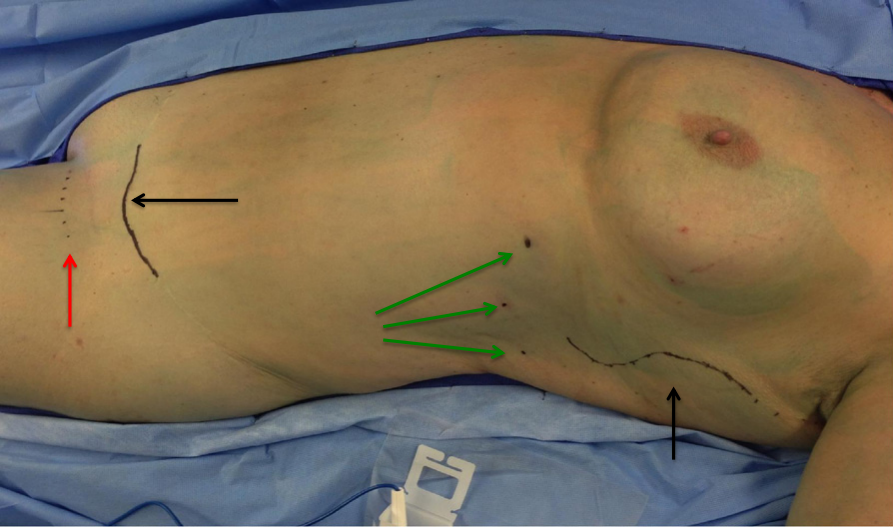
**Patient with a history of vulvar carcinoma who has undergone superficial lymphadenectomy and resulting lower extremity lymphedema.** Incision design for elevation of vascularized lymph node flap based on thoracodorsal vessels and incision design for abdominal incision for inset of flap (black arrows). Green arrows indicate sites of injection of indocyanine green to identify lymph nodes draining the chest for inclusion in the flap. Red arrow indicates the site for inset of the vascularized lymph node flap above the muscular fascia in the region of superficial lymphadenectomy. The patient had undergone technetium injection into the left hand the day prior to surgery in order to identify lymph nodes draining this extremity ('reverse lymphatic mapping’).

**Figure 7:**
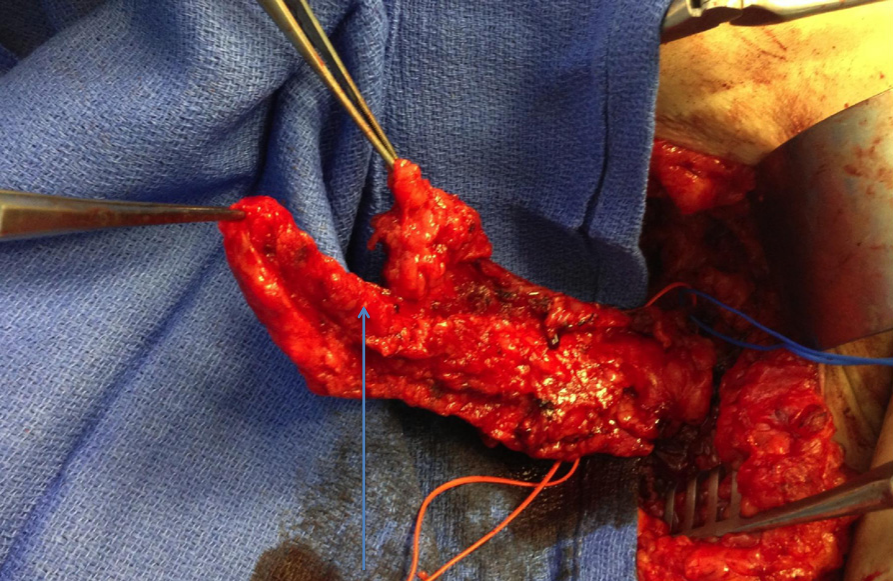
**Intraoperative view of lymph node flap based on thoracodorsal artery and vein.** The picture depicts the lower chest thoracic lymph nodes located at Level III axillary lymph node position. These lymph nodes did not take up technetium, thereby avoiding the extremity drainage basin.

The most recent advancement in surgical lymphedema treatment is vascularized lymph node transfer. Healthy lymph nodes, artery and vein are transplanted from an unaffected axilla, groin or cervical/neck to the affected area of lymphedema [141-145] (Figures 3, 4, 5, 6, 7). One hypothesis is that the transferred lymph nodes act as a pump and suction pathway for lymphatic clearance [142]. Lin *et al*. report, at more than four years post-operation, a 51% reduction in upper extremity volume when transferring groin lymph nodes to the wrist [142]. Cheng *et al*. report a reduction in circumference of more than 60% in the lower extremity when utilizing submental lymph nodes transferred to the ankle [141]. In Becker’s study, of 24 patients with groin lymph node transfer to the axilla for upper extremity lymphedema, physiotherapy was able to be discontinued in 62.5% of patients [145].

Development of lymphedema in the donor site remains a concern. Initial literature demonstrated morbidity to be minimal [143]. Recent reports suggest a higher incidence of secondary lymphedema than was previously considered. In studying patients who had undergone vascularized lymphatic transfer, Vignes *et al*. found that six of 26 patients developed chronic lymphedema, defined as ≥2 cm difference versus the contralateral side [146]. This donor site lymphedema remains a significant concern and any transfer of VLNs (vascularized lymph nodes) must include an evaluation of lymph nodes draining the donor site.

Reverse lymphatic mapping is a technique that has been suggested to differentiate the lymph node basins draining an extremity from those that may be useful for vascularized lymph node harvest and transfer [147]. The technique is similar to the axillary reverse mapping that has been presented by Klimberg *et al*. [148]. Reverse lymphatic mapping builds on the principle of differential identification of lymph node basins with separate drainage patterns. Technetium can be injected into the dorsal webspace of an extremity similar to the injection technique that is performed during lymphoscintigraphy. Simultaneously, lymphazurin blue may be injected into either trunk or groin nodal basins. By differentiating lymph nodes that drain an extremity from those amenable to vascularized lymph node transfer, this technique may reduce the incidence of secondary lymphedema at the donor site.

## Conclusions

The management of lymphedema remains a complex entity that requires knowledge of the underlying pathophysiology. Patients with breast or gynecologic malignancy are at risk for the development of lymphedema, particularly in the setting of lymphadenectomy and/or radiation treatment. Although management with physical therapy remains the primary treatment, exciting new surgical treatments are under active investigation.

## Consent

Written informed consent was obtained from each patient from whom images have been reproduced into the main body of this manuscript.

## Abbreviations

ALND: Axillary lymph node dissection; BMI: Body mass index; CDPT: Complex decongestive physiotherapy; LEL: Lower extremity lymphedema; MLD: Manual lymphatic drainage; PLND: Pelvic lymph node dissection; SNP: Single nucleotide polymorphism; UEL: Upper extremity lymphedema; VLN: Vascularized lymph node.

## Competing interests

The authors declare that they have no competing interests.

## Authors’ contributions

PT was responsible for the overall review paper design, writing and editing of all drafts of the manuscript, and has approved the final version of the submitted manuscript. RS was involved in writing and editing portions of the manuscript and has approved the final version of the submitted manuscript. MC was involved in writing and editing portions of the manuscript and has approved the final version of the submitted manuscript. SPP was involved in writing and editing of all drafts of the manuscript. All authors read and approved the final manuscript.
